# The Relationship between Inspiratory Muscle Strength and Cycling Performance: Insights from Hypoxia and Inspiratory Muscle Warm-Up

**DOI:** 10.3390/jfmk9020097

**Published:** 2024-05-31

**Authors:** André Luiz Musmanno Branco Oliveira, Gabriel Dias Rodrigues, Philippe de Azeredo Rohan, Thiago Rodrigues Gonçalves, Pedro Paulo da Silva Soares

**Affiliations:** 1 Department of Physiology and Pharmacology, Fluminense Federal University, Rua Prof. Hernani Pires de Melo 101, Bloco E-217, Niterói 24210-130, RJ, Brazil; andremusmanno@gmail.com (A.L.M.B.O.); philipperohan@id.uff.br (P.d.A.R.); tr.goncalves@yahoo.com.br (T.R.G.); ppssoares@id.uff.br (P.P.d.S.S.); 2 Department of Clinical Sciences and Community Health, University of Milan, 20122 Milan, Italy

**Keywords:** respiratory muscles, ventilation, elevated altitude, exercise performance, cycling, hypoxia

## Abstract

Hypoxia increases inspiratory muscle work and consequently contributes to a reduction in exercise performance. We evaluate the effects of inspiratory muscle warm-up (IMW) on a 10 km cycling time trial in normoxia (NOR) and hypoxia (HYP). Eight cyclists performed four time trial sessions, two in HYP (FiO_2_: 0.145) and two in NOR (FiO_2_: 0.209), of which one was with IMW (set at 40% of maximal inspiratory pressure—MIP) and the other was with the placebo effect (PLA: set at 15% MIP). Time trials were unchanged by IMW (NOR_IMW_: 893.8 ± 31.5 vs. NOR_PLA_: 925.5 ± 51.0 s; HYP_IMW_: 976.8 ± 34.2 vs. HYP_PLA_: 1008.3 ± 56.0 s; *p* > 0.05), while ventilation was higher in HYP_IMW_ (107.7 ± 18.3) than HYP_PLA_ (100.1 ± 18.9 L.min^−1^; *p* ≤ 0.05), and SpO_2_ was lower (HYP_IMW_: 73 ± 6 vs. HYP_PLA_: 76 ± 6%; *p* ≤ 0.05). A post-exercise-induced reduction in inspiratory strength was correlated with exercise elapsed time during IMW sessions (HYP_IMW_: r = −0.79; *p* ≤ 0.05; NOR_IMW_: r = −0.70; *p* ≤ 0.05). IMW did not improve the 10 km time trial performance under normoxia and hypoxia.

## 1. Introduction

High-intensity cycling time trial tests increase respiratory demand and generate diaphragm fatigue [1], activating the respiratory muscle metaboreflex, leading to peripheral muscle vasoconstriction, and reducing exercise tolerance [2]. Not surprisingly, compared to normoxia, a hypoxic condition demands further respiratory work [3], exacerbating respiratory fatigue at the same workload of locomotor muscles [4] and even during exercise at a matched ventilatory level [5]. Several studies investigated strategies to improve respiratory muscles performance under hypoxia, enhancing exercise time trial and tolerance to fatigue through inspiratory muscle training (IMT) [6,7,8] or respiratory muscle unloading [3]. However, to date, no other simple methods are available to produce acute effects for respiratory muscles with practical applications for athletes exercising under hypoxia.

In normoxic conditions, the inspiratory muscle warm-up (IMW) as a preparatory strategy before exercise has been employed to enhance performance, showing favorable results [9,10,11,12,13]. However, controversial results showed no IMW improvements in cycling [14,15], even in exhaustive time trial cycling [16]. These contradictory results may be due to either a short exercise task [14,15] that may not be enough to generate diaphragmatic fatigue or a longer distance cycling in normoxic conditions [16] that only occasionally generates a robust respiratory demand, making it harder to observe any detectable effect of IMW. Conversely, cycling under hypoxia can overload inspiratory muscles [3], which might be a factor in limiting exercise performance in cycling competitions at high altitudes. Therefore, it is plausible that IMW may offer an effective ergogenic resource capable of enhancing cycling performance under hypoxia. The potential IMW application for exercise performance improvements in hypoxic conditions remains unexplored.

IMW promotes acute improvements in inspiratory muscle strength [9,15,17,18] and may delay the activation of respiratory metaboreflex [14]. Taken together, it is reasonable that these IMW effects could increase exercise tolerance in hypoxic environments. Thus, understanding the role of IMW in cycling performance under hypoxia may offer new insights into its isolated usage or in combination with other strategies in cyclists’ practice at high altitudes.

Therefore, we hypothesize that IMW will enhance exercise performance under hypoxia, which should be associated with an attenuated post-exercise drop in inspiratory muscle strength. The current study aimed to evaluate the effects of IMW on cycling time trial performance in hypoxia.

## 2. Materials and Methods

### 2.1. Participants

Eight male well-trained cyclists (age: 32 ± 7 years; height: 1.78 ± 0.1 m; body mass: 73.5 ± 7.4 kg; VO_2_max: 5.0 ± 0.6 L·min^−1^; mean ± SD) participated in this study. Cyclists were advised to avoid caffeine, alcohol, and moderate to intense exercise 24 h before the visits. All the cyclists in this study were in the base phase, meaning they were at the initial stage of a cyclist’s season, and their cycling experience is approximately 5 years and a typical training regimen of 240 to 350 km per week. Exclusion criteria were the use of performance-enhancing drugs or supplements and any concomitant disease. The Institutional Ethics Committee approved the present study (1.252.971/2015), and each cyclist signed an informed consent before participating.

### 2.2. Procedures

**Figure 1:**
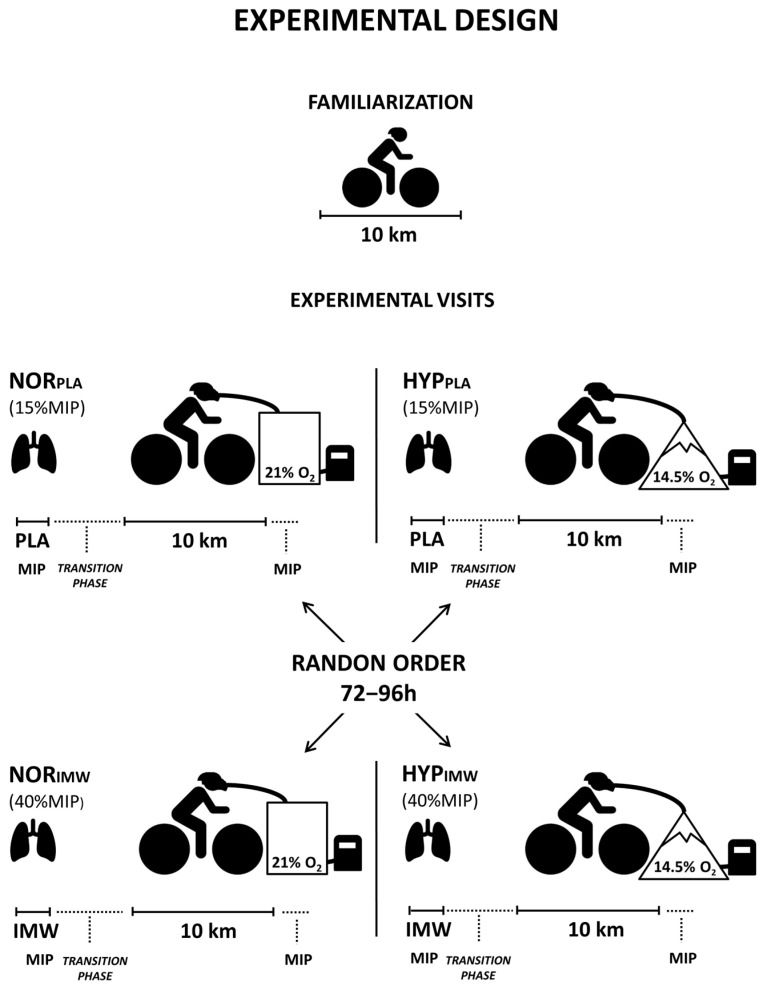
As shown in the experimental design, the cyclists performed a familiarization with a 10 km time trial on the first visit. On other days, the four experimental visits were randomized, but the cyclists were unaware of the gas condition in the mask and the inspiratory warm-up load.

The experiments in this randomized and placebo-controlled study were completed in five visits separated by 72–96 h. In the first visit, body mass, height, maximal inspiratory pressure (MIP: 155 ± 37 cm H_2_O), and pulmonary function tests were measured (forced vital capacity: 5.2 ± 0.6 L; forced expiratory volume in 1s: 4.0 ± 0.6 L). Then, the cyclists performed a 10 km cycling time trial for individual adaptation to the test protocol. Visual feedback provided by the cycle ergometer monitor was allowed only during this familiarization session. During the following four visits, a 10 km time trial was conducted with a prior effective IMW or placebo (PLA), both in normoxia (FiO_2_ = 0.209; ≈162 m) or hypoxia (FiO_2_ = 0.145; ≈3085 m—equivalent altitude) in random order (Figure 1).

### 2.3. Materials

The pulmonary tests were evaluated via spirometry (MRS/DATALINK, Montpellier, France), and MIP was assessed by an inspiratory device (POWERBreathe KH2, HaB International Ltd., Southam, UK). During all visits, a two-way respiratory valve (Y2730, Hans Rudolph, Inc., Shawnee, OK, USA) was linked to the air outlet of the pneumotachograph through a customized adapter to allow the ventilatory data recordings (VO2000, MedGraphics, Saint Louis, MO, USA) together with the hypoxic machine (Everest Summit II, Hypoxico, NY, USA). For recording cardiac variables, non-invasive electrical impedance cardiography was used (Physioflow^®^ LAB1TM, Hemodynamics Redefined, Manatec Biomedical, Folschviller, France). The arterial oxygen saturation (SpO_2_) values were assessed through a finger pulse oximeter (Oxy-Go QuickCheckTM PRO, Oxymeter Plus, Inc., Roslyn Heights, NY, USA). Cycling time trial tests were performed on a magnetic and air resistance cycle ergometer (Wattbike PRO, Wattbike Ltd., Nottingham, UK).

### 2.4. Familiarization with Time Trial and IMW

In the first visit, a 10 km time trial test was conducted for familiarization with the protocol. This procedure avoids the possibility of self-paced cycling performance learning on subsequent visits [16,19]. After the time trial, the cyclists were introduced to the IMW technique and received instructions about the correct use of diaphragmatic breathing and the respiratory rate established by the metronome.

### 2.5. Time Trial Tests

In the four succeeding experimental visits, MIP was determined one hour before the tests, and the cyclists were instrumented with electrodes. Then, cyclists executed IMW or PLA, and immediately after, a face mask was positioned for ventilatory measurements. The cyclists were unaware of the gas mixture and the load from the inspiratory device during the experiment. The elapsed time and power output were inaccessible to cyclists, but they could observe the cadence and the remaining distance during time trials. The air resistance of the cycle ergometer and cadence during the time trial tests were self-selected by each cyclist, but they were not allowed to lift from the seat. During all time trial tests, cyclists were encouraged to complete 10 km as fast as possible. To test the isolated effect of IMW on performance, whole-body warm-ups were not allowed. At 2.5 km intervals, the dyspnea and peripheral muscles’ perceptual effort were registered using Borg’s scales CR10 and RPE 6–20 [20], respectively, and the SpO_2_ was also recorded.

### 2.6. Inspiratory Muscle Pressure

Maximal inspiratory pressure was determined as the highest value of the three reproducible measurements, which had a variation of less than 5% among them [21]. This procedure was repeated every visit 1 h before the time trial tests, and the inspiratory load prescription used for IMW and PLA was calculated based on the MIP value recorded at the first visit. Furthermore, another MIP maneuver was done immediately after exercise to quantify the acute post-exercise-induced reduction in inspiratory strength.

### 2.7. Inspiratory Muscle Warm-Up and Placebo

All cyclists remained seated on the cycle ergometer during the inspiratory warm-up maneuvers. The IMW and PLA sessions were recorded using a digital inspiratory device (POWERBreathe KH2, HaB International Ltd., Southam, UK). The cyclists performed inspiratory maneuvers in ambient air before wearing the gas condition mask. The protocol consisted of two sets of 30 inspiratory maneuvers set at 40% MIP for IMW and 15% for PLA, with a 60-s recovery between sets [9,15,18]. A metronome marked the respiratory rate at 15 cycles/min during the IMW or PLA in all the visits, while the variables were recorded by ad hoc software (Breathe-Link KH2 Medic 1.0c, Southam, UK).

### 2.8. Statistical Analysis

Data normality was analyzed using the Shapiro–Wilk test (see Supplementary Materials, Table S1). Herein, the same individuals performed all visits and there were two factors to be analyzed as follows: (1) both oxygen conditions (normoxia and hypoxia) and (2) both warm-up conditions (IMW and PLA). Thus, we chose a two-way analysis of variance (ANOVA) with repeated measures in both factors (FiO_2_ and warm-up) to compare time trial performance, perceptual, and physiological variables. Regarding inspiratory warm-up variables among visits (i.e., pressure, power, volume, and flow during IMW or PLA) we decided on a one-way ANOVA since the inspiratory warm-ups were always performed in ambient air. After, the Bonferroni post-hoc test was carried out to determine where the differences were. Additionally, the paired *t*-test was used to compare MIP pre- vs. post-exercise and total average VE in HYP_PLA_ vs. VE in HYP_IMW_. Statistical difference was considered when α ≤ 0.05. Results are described as mean and standard deviation. To calculate the effect size (es), we used Cohen’s d and f indexes when appropriate (G*power v3.1.9.2; Heinrich-Heine-Universität, Düsseldorf, Germany) and the values were classified as trivial (0–0.2), small (0.2–0.6), moderate (0.6–1.2), large (1.2–2.0), and very large (2.0–4.0) [22]. Pearson’s correlation was used to evaluate the relationship between exercise time and MIP variation post-exercise and the regression line was plotted. Statistical analysis was done using specific software (GraphPad Prism v6.0, La Jolla, CA, USA).

## 3. Results

### 3.1. Time Trial Performance

**Figure 2:**
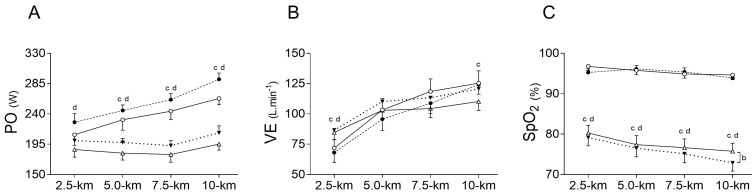
Comparison of power output (PO) (**A**), oxygen saturation (SpO_2_) (**B**), and ventilation (VE) (**C**) between conditions during time trial. NOR_PLA_; open circle (〇); NOR_IMW_; closed circle (⬤), HYP_PLA_; open triangle (△) and HYP_IMW_; closed inverted triangle (▼). Statistical difference: Differences between NOR_PLA_ vs. NOR_IMW_. (b) Differences between HYP_PLA_ vs. HYP_IMW_. (c) Differences between NOR_PLA_ vs. HYP_PLA_. (d) Differences between NOR_IMW_ vs. HYP_IMW_. Bonferroni’s post-hoc test. Mean ± SE.

Inspiratory muscle warm-up did not change exercise elapsed time compared to PLA in both, normoxia (NOR_IMW_: 893.8 ± 31.5 s vs. NOR_PLA_: 925.5 ± 51.0 s), and hypoxia (HYP_IMW_: 976.8 ± 34.2 s vs. HYP_PLA_: 1008.3 ± 56.0 s); (*p* = 0.151; es = 0.61). Likewise, IMW was unable to statistically change the power output in both normoxia (NOR_IMW_: 255 ± 23 W vs. NOR_PLA_: 236 ± 35 W) and hypoxia (HYP_IMW_: 200 ± 19 W vs. HYP_PLA_: 186 ± 26 W); (PO_MEAN_: *p* = 0.147; es = 0.62). On the other hand, compared to normoxia, under hypoxia, the time trial test was slower, increasing the elapsed time to complete the test (*p* < 0.001; es = 2.18), and PO_MEAN_ decreased (*p* = 0.001; es = 2.04). There was no interaction between IMW and hypoxia for exercise time (*p* = 0.973; es = 0.03) nor for PO_MEAN_ (*p* = 0.696; es = 0.15) (Figure 2).

### 3.2. Physiological Responses

Higher values of ventilation (VE) were found at 2.5 km during hypoxic sessions compared to normoxic sessions (*p* = 0.001; es = 2.13), while IMW did not change VE (*p* = 0.75; es = 0.13), and there was no interaction between factors (*p* = 0.23; es = 0.50). Moreover, toward the last segment of the exercise (10 km), higher values of VE were found during NOR_PLA_ compared to HYP_PLA_ (*p* = 0.05; es = 0.86), but there was no difference between NOR_IMW_ and HYP_IMW_ (*p* = 0.45; es = 0.28) (Figure 2). In addition, the total average VE was higher during HYP_IMW_ (107.7 ± 18.3 L·min^−1^) compared to HYP_PLA_ (100.1 ± 18.9 L·min^−1^; *p* = 0.03; es = 1.00). Furthermore, as expected, SpO_2_ values were reduced by hypoxia compared to normoxia (*p* < 0.0001; es = 4.90). Nevertheless, in hypoxia, lower values of SpO_2_ were found at 10 km of the HYP_IMW_ session compared to HYP_PLA_ (*p* = 0.04; es = 1.08) (Figure 2).

**Figure 3:**
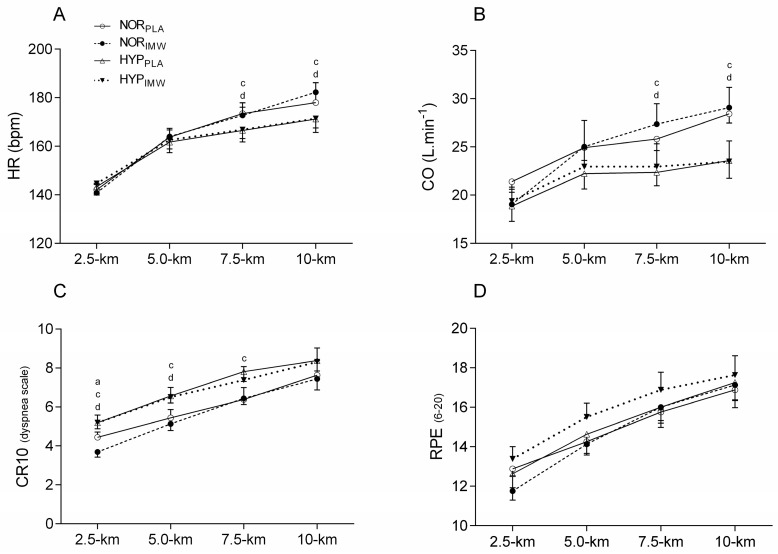
Comparison of heart rate (HR), cardiac output (CO), and rating of perceived effort for dyspnea (CR10) and for peripheral muscles (RPE). NOR_PLA_; open circle, NOR_IMW_; closed circle, HYP_PLA_; open triangle and HYP_IMW_; closed inverted triangle. (**A**) Differences between NOR_PLA_ vs. NOR_IMW_; (**B**) Differences between HYP_PLA_ vs. HYP_IMW_. (**C**) Differences between NOR_PLA_ vs. HYP_PLA_; (**D**) Differences between NOR_IMW_ vs. HYP_IMW_. Bonferroni’s post-hoc test. Mean ± SE. Values of five cyclists for HR and CO, the others were excluded due to technical problems during acquisition data. Statistical difference: (a) Differences between NOR_PLA_ vs. NOR_IMW_. (c) Differences between NOR_PLA_ vs. HYP_PLA_. (d) Differences between NOR_IMW_ vs. HYP_IMW_. Bonferroni’s post-hoc test. Mean ± SE.

Concerning heart rate (HR), stroke volume (SV), and CO during the exercises, lower values of HR and CO were found during 7.5 km (HR; *p* = 0.01; es = 2.27) (CO; *p* = 0.04; es = 1.56) and 10 km (HR; *p* = 0.008; es = 2.44) (CO; *p* = 0.004; es = 3.00) in hypoxic sessions, but no change was found when using IMW (*p* > 0.05) (Figure 3). No differences were found in the average SV between conditions during time trial tests (SV; NOR_PLA_: 151 ± 14 mL; NOR_IMW_: 146 ± 24 mL; HYP_PLA_: 136 ± 26 mL; HYP_IMW_: 138 ± 31 mL; *p* = 0.25; es = 0.68).

The rating of perceived effort for dyspnea (CR10) was higher during hypoxic conditions at 2.5 km (*p* = 0.003; es = 1.73), 5 km (*p* = 0.002; es = 1.74), 7.5 km (*p* = 0.012; es = 1.26) and 10 km (*p* = 0.029; es = 1.03). During NOR_IMW_, the dyspnea score was lower than during NOR_PLA_ at 2.5 km (*p* = 0.048; es = 1.00). However, there was no interaction between hypoxia and IMW for dyspnea. There were no statistical differences in RPE during the time trial between conditions (*p* > 0.05) (Figure 3).

### 3.3. Inspiratory Strength and Exercise Time Relationships

**Figure 4:**
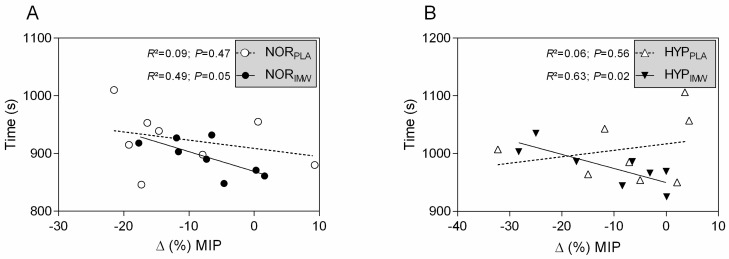
(**A**,**B**) Pearson’s correlation of exercise time and variation of maximal inspiratory pressure post-exercise. NOR_PLA_; open circle, NOR_IMW_; closed circle, HYP_PLA_; open triangle and HYP_IMW_; closed inverted triangle. Linear regression lines equations: NOR_PLA_: Y = −1.426X + 909; NOR_IMW_: Y = −3.425X + 869; HYP_PLA_: Y = 1.115X + 1017; HYP_IMW_: Y = −2.439X + 949.8.

As expected, during the inspiratory warm-up sessions, the IMW generated greater inspiratory power and mouth pressure than the PLA conditions (see Table 1). However, the flow and volume were not different. Also, the post-exercise fall in inspiratory strength occurred in all sessions, and IMW did not attenuate it (see Table 2). However, it is interesting that in IMW, in both normoxia and hypoxia, the changes in inspiratory strength after exercise were negatively correlated with exercise elapsed time to complete 10 km (see Figure 4; HYP_IMW_: r = −0.79; r^2^ = 0.63; *p* = 0.02; and NOR_IMW_: r = −0.70; r^2^ = 0.49; *p* = 0.05). However, it did not happen in PLA conditions (HYP_PLA_: r = 0.24; r^2^ = 0.06; *p* = 0.56; and NOR_PLA_: r = −0.30; r^2^ = 0.09; *p* = 0.47).

## 4. Discussion

Our main findings were as follows: (1) IMW did not improve 10 km cycling time trial performance during normoxia and hypoxia; (2) during HYP_IMW_, the average VE was increased compared to the HYP_PLA_ session, while SpO_2_ was lower in the last segment, and these differences were not observed during normoxic sessions, and (3) correlation analysis showed an inverse relation between exercise elapsed time during the time trial and the fall in inspiratory strength, which curiously only occurred in IMW sessions both in normoxia and hypoxia. In IMW sessions, the cyclists who had less of a fall in inspiratory muscle strength after exercise tended to be the same ones who had faster time trial performances (Figure 4).

### 4.1. Effects of IMW in Normoxic Exercise

Similarly to the current investigation, a previous study that used IMW before a 10 km cycling time trial in normoxia did not find performance improvements [16]. In the study by Johnson and co-authors [16], the period between the end of IMW and the time trial test was about 22 min, and a cycling warm-up (~15 min) was included in this transition phase before the time trial test. Differently, in our investigation, the transition phase duration between IMW and the time trial was about nine minutes, and cycling warm-up was not allowed. According to Hawkes and co-authors [17], IMW generates a transient increase in inspiratory strength that decreases to baseline values after 15 min. Therefore, a shorter transition phase was done between IMW and the time trial to avoid a possible loss of or reduction in IMW’s effect. However, such methodological differences in the present investigation did not warrant performance gain during the normoxic 10 km time trial test. In contrast, various intense short-term exercises (i.e., ≤6 min task) are enhanced with IMW, such as the 6-min all-out rowing task [9], the 30-s anaerobic Wingate’s test [12], and exhaustive intermittent run tolerance [10]. However, no improvement was found in intermittent cycling activities [14,15]. Therefore, in normoxic conditions, IMW seems to be useful for several high-intensity and short-time exercise activities.

In normoxia, IMW did not change the ventilatory levels during time trial exercise (Figure 2B), corroborating previous studies [16,23]. However, perceived dyspnea was lower in NOR_IMW_ than in NOR_PLA_, but only during the first segment of the time trial (0–2.5 km) and without changes in the rating of perceived effort for locomotor muscles. Some studies have found that IMW reduced the perceived dyspnea [9,11], while others did not find differences [14,23]. These controversial results can be in part explained by the differences in experimental design and exercise modalities, which can be cycling [14], rowing [9], running [10], and others [11,23]. Despite the limitations, IMW is a potential strategy for reducing perceived dyspnea in some exercise conditions, but the mechanisms remain unclear.

### 4.2. Effects of IMW in Hypoxic Exercise

In contrast to our hypothesis, our preliminary data suggest that IMW did not improve time trial performance under hypoxia. Before raising the possibility that IMW is ineffective under hypoxia, we attribute the absence of performance effect to two main reasons: (1) it is known that athletes have an attenuated diaphragmatic metaboreflex response [24], thereby reducing the possibility of the acute effects of IMW, making it harder to identify this mechanism compared to sedentary individuals who present higher diaphragmatic metaboreflex activation; and (2) in time trial tests, the cyclists regulate their workload pace, avoiding fatigue to enable them to complete the test. For this reason, it is plausible that in a 10 km time trial under hypoxia, the cyclists did not sustain a strenuous heavy-intensity exercise, generating an accentuated respiratory fatigue and its consequences. This reasoning can be reinforced by observing the data in Table 2, where the MIP variations post-exercise decline similarly in normoxia and hypoxia, probably because the cyclists regulated their workload at a level significantly lower under hypoxia.

In the last segment of the time trial test (i.e., 7.5–10 km) in HYP_IMW_, the SpO_2_ was lower, while the average of VE was ~10 L·min^−1^ higher than HYP_PLA_. Since the present study is the first to evaluate IMW as a strategy to improve exercise under hypoxia, the following discussion will consider studies that used IMT. Therefore, no direct comparisons are possible since different approaches were employed. Thus, with the use of IMT, Hursh and colleagues [8] observed an increase in VE, oxygen uptake, and a faster time trial performance (i.e., 1.4%, ~33 s) during 20 km in hypoxia (FiO_2_ = 0.16) [8]. Applying IMT, Salazar-Martínz and co-authors [7] have also found improvements in exercise performance under hypoxia, but such results are not always observed in other studies [6]. On the other hand, IMT seems to reduce the physiological demand in submaximal exercise under hypoxia [25]. Differences in exercise tasks may have influenced the different results. In our results, IMW conditions had a discreet average time trial advantage but not enough to improve exercise performance consistently.

The higher ventilatory response in time trial under hypoxia with the use of IMW observed here was also found by Hursh and colleagues [8] investigation using IMT. Despite the similar results for ventilation, the mechanisms involved in muscle training are different from the muscle warm-up. Whereas a training process (i.e., IMT) involves structural and neural adaptations in inspiratory muscles, the IMW involves acute responses that, according to Hawkes and colleagues [17], increase transiently inspiratory force output, which might be related to an altered central drive, allowing a better inspiratory muscle synergy. Thus, IMW with a higher stimulus from peripheral chemoreceptors, taken together, could sustain higher ventilation under hypoxic conditions. These pathways can reasonably explain the discreet additional sustained ventilatory response. On the other hand, the exact mechanisms that led to a lower SpO_2_ response during HYP_IMW_ compared to HYP_PLA_ are unknown.

Regarding cardiovascular data, heart rate (HR) and cardiac output (CO) were unchanged by IMW, but lower values were found during hypoxic time trial sessions than those in normoxia (Figure 3A,B). At first glance, these results contrast with previous studies interestingly reviewed by Siebenmann and Lundby [26], which reported higher HR and CO during acute hypoxic submaximal exercise. However, as observed here, the self-selected exercise loads were significantly lower in hypoxic sessions (Figure 2A), which may partially explain the lower values of HR and CO in hypoxic exercise. Naturally, the different workloads on the cycle ergometer between normoxia and hypoxia may mask the expected difference when the workload is the same for both conditions, where hypoxia would require higher CO. Moreover, it was only in normoxic conditions that the cyclists felt capable of increasing their workloads, especially in the last segment (7.5–10 km) of the time trial test, which consequently demanded higher CO from the middle to the end of the exercise test.

### 4.3. Time Trial Performance and the Post-Exercise Inspiratory Muscle Strength Decline

An important finding in our study is the inverse relationship between exercise elapsed time and post-exercise inspiratory strength decline. Thus, the lower decline in inspiratory muscle strength after exercise correlates with faster time trials. Such an effect is only observed in IMW sessions, independently of the inspired oxygen condition, suggesting an exclusive effect of IMW. Although the decline of MIP post-exercise is frequently reported [9,15,27], to our knowledge, no studies showed such IMW effect individually related to exercise performance. These relationships suggest that in IMW sessions, the MIP variations may be useful for coaches to identify the cyclists’ inspiratory training necessity (e.g., when a cyclist has more decline in MIP and is slower within the team), however, no direct benefit in performance was identified with IMW. The mechanisms underlying this relationship are unknown; however, it is possible to speculate some possible pathways as follows: (1) IMW provides a better synergy among respiratory muscles [17] and probably induces the post-activation potentiation mechanism, increasing inspiratory muscle strength. Thus, the inspiratory muscle work was probably optimized by IMW, subjectively leading to more self-confidence regarding incrementing the workload during the time trial. (2) Perhaps, with more inspiratory strength by IMW, the phrenic afferent inputs to the central nervous system during exercise may not early trigger the diaphragmatic metaboreflex, thus making linear the fine-tuning relationship between respiratory and locomotor muscles during the time trial test. Further investigations are needed to evaluate the possible mechanisms involved in IMW on inspiratory muscles’ strength and locomotor muscles’ performance.

### 4.4. Future Directions

Our preliminary data suggest that IMW does not affect exercise performance under hypoxia. Future studies should consider testing the IMW effect on other exercise modalities, as well as on different hypoxic levels and other IMW protocols. We encourage future studies to investigate inter-individual responsiveness to IMW and identify potential responders and non-responders, which is valuable to practical applicability for cycling competitions. Moreover, considering that IMW promotes fine-tuning in the relationship between exercise time and post-exercise inspiratory strength fall (Figure 4), we encourage future studies to investigate how the feedback relation between inspiratory muscles and self-paced works. Not less importantly, future directions should focus on the mechanisms underlying the IMW to answer why the strategy may or may not work. Thus, additional measurements for inspiratory and locomotor muscle oxygenation, arterial gasometry, inspiratory fatigue tests, and metabolic analysis should be considered.

### 4.5. Limitations

This study has several limitations that may be considered for a better interpretation of the results. First, we did not evaluate respiratory and locomotor muscle oxygenation and, for technical reasons, neither did metabolic gas exchanges during time trial tests. We recognize that the absence of these data limits our interpretation of the effect of IMW on oxygen consumption pathways. However, the present investigation focused on the effect of IMW on exercise performance under hypoxic conditions. Second, our results cannot be extrapolated to all athletes and modalities. Therefore, this issue needs further investigation. Third, the hypoxic environment was simulated in normobaric conditions, which is different from a hypobaric altitude environment. Thus, future research with a similar approach should focus on a natural high-altitude environment or the use of a hypobaric chamber. Finally, despite the limitations, time trial tests are largely used in cycling training and are similar to real competitions. Therefore, a potential application of the IMW is optional and can be considered for cycling training and competing at sea level and moderate altitude.

## 5. Conclusions

Our preliminary findings show that the traditional IMW protocol does not enhance 10 km time trial performance in normoxia and hypoxia but does reveal a relationship between exercise performance and the post-exercise decline in inspiratory muscle strength. Under hypoxia, IMW increased average ventilation during the cycling time trial, which did not occur in normoxia but reduced dyspnea sensation at the beginning of the normoxic exercise. Thus, further investigations are necessary to elucidate the mechanisms and potential applications of IMW for exercise tasks in sea-level and high-altitude environments.

## Figures and Tables

**Table 1:** Inspiratory variables during inspiratory muscle warm-up sessions.

IMW Indexes	NOR_PLA_	NOR_IMW_	HYP_PLA_	HYP_IMW_	es NOR_IMW_	es HYP_IMW_
Pressure _(cmH2O)_	15.16 ± 3.18	39.84 ± 7.96 *	15.76 ± 4.16	40.09 ± 8.65 ^#^	(5.04)	(4.54)
Power _(W)_	3.86 ± 1.91	11.09 ± 5.62 *	4.47 ± 3.78	12.27 ± 5.42 ^#^	(1.83)	(2.97)
Volume _(L)_	2.92 ± 0.56	3.27 ± 0.78	2.90 ± 0.79	2.94 ± 0.80	(0.44)	(0.05)
Flow _(L/s)_	2.32 ± 0.66	2.48 ± 0.76	2.50 ± 1.34	2.80 ± 0.68	(0.27)	(0.31)

The values of inspiratory muscle warm-up indexes presented as mean ± SD of each session. Cohen’s d was used as effect size (es) for IMW vs. PLA. *, *p* ≤ 0.05 compared to NOR_PLA_; ^#^, *p* ≤ 0.05 compared to HYP_PLA_.

**Table 2:** Maximal inspiratory pressures in every visit pre- and post-exercise.

	NOR_PLA_	NOR_IMW_	HYP_PLA_	HYP_IMW_
MIP values				
MIP_PRE (cmH2O)_	135 ± 34	149 ± 34	146 ± 37	149 ± 36
MIP_POST-EXER (cmH2O)_	120 ± 30 * (1.06)	138 ± 35 * (1.00)	134 ± 34 (0.56)	132 ± 36 * (0.89)
MIP changes				
∆MIP_POST-PRE (%)_	−11 ± 11	−7 ± 6	−8 ± 12	−11 ± 11

The values are presented as mean ± SD. Maximal inspiratory pressure before (MIP_PRE_) vs. after exercise (MIP_POST-EXER_). The variation (∆) of MIP (post-exercise–pre) expressed as a percentage (%). The symbol * is equal to *p* ≤ 0.05 compared to MIP_PRE_. Cohen’s (d) was used as effect size.

## Data Availability

Raw data may be available upon a reasonable written request to the first and the corresponding author.

## Supplementary Materials

The following supporting information can be downloaded at: https://www.mdpi.com/article/10.3390/jfmk9020097/s1. Table S1: Shapiro-Wilk Test *p*-values results.

## References

[1] Wüthrich T.U., Eberle E.C., Spengler C.M. (2014). Locomotor and diaphragm muscle fatigue in endurance athletes performing time trials of different durations. Eur. J. Appl. Physiol..

[2] Dempsey J.A., Romer L., Rodman J., Miller J., Smith C. (2006). Consequences of exercise-induced respiratory muscle work. Respir. Physiol. Neurobiol..

[3] Amann M., Pegelow D.F., Jacques A.J., Dempsey J.A. (2007). Inspiratory muscle work in acute hypoxia influences locomotor muscle fatigue and exercise performance of healthy humans. Am. J. Physiol. Regul. Integr. Comp. Physiol..

[4] Babcock M.A., Johnson B.D., Pegelow D.F., Suman O.E., Griffin D., Dempsey J.A. (1995). Hypoxic effects on exercise-induced diaphragmatic fatigue in normal healthy humans. J. Appl. Physiol..

[5] Vogiatzis I., Georgiadou O., Koskolou M., Athanasopoulos D., Kostikas K., Golemati S., Wagner H., Roussos C., Wagner P.D., Zakynthinos S. (2007). Effects of hypoxia on diaphragmatic fatigue in highly trained athletes. J. Physiol..

[6] Downey A.E., Chenoweth L.M., Townsend D.K., Ranum J.D., Ferguson C.S., Harms C.A. (2007). Effects of inspiratory muscle training on exercise responses in normoxia and hypoxia. Respir. Physiol. Neurobiol..

[7] Salazar-Martínez E., Gatterer H., Burtscher M., Orellana J.N., Santalla A. (2017). Influence of Inspiratory Muscle Training on Ventilatory Efficiency and Cycling Performance in Normoxia and Hypoxia. Front. Physiol..

[8] Hursh D.G., Baranauskas M.N., Wiggins C.C., Bielko S., Mickleborough T.D., Chapman R.F. (2019). Inspiratory Muscle Training: Improvement of Exercise Performance With Acute Hypoxic Exposure. Int. J. Sports Physiol. Perform..

[9] Volianitis S., McConnell A.K., Koutedakis Y., Jones D.A. (2001). Specific respiratory warm-up improves rowing performance and exertional dyspnea. Med. Sci. Sports Exerc..

[10] Tong T.K., Fu F.H. (2006). Effect of specific inspiratory muscle warm-up on intense intermittent run to exhaustion. Eur. J. Appl. Physiol..

[11] Lin H., Kwokkeung T.T., Huang C., Nie J., Lu K., Quach B. (2007). Specific inspiratory muscle warm-up enhances badminton footwork performance. Appl. Physiol. Nutr. Metab..

[12] Özdal M., Bostanci O., Dağlioğlu O., Ağaoğlu S.A., Kabadayi M. (2016). Effect of respiratory warm-up on anaerobic power. J. Phys. Ther. Sci..

[13] Cirino C., Marostegan A.B., Hartz C.S., Moreno M.A., Gobatto C.A., Manchado-Gobatto F.B. (2023). Effects of Inspiratory Muscle Warm-Up on Physical Exercise: A Systematic Review. Biology.

[14] Cheng C., Tong T.K., Kuo Y., Chen P., Huang H., Lee C. (2013). Inspiratory muscle warm-up attenuates muscle deoxygenation during cycling exercise in women athletes. Respir. Physiol. Neurobiol..

[15] Ohya T., Hagiwara M., Suzuki Y. (2015). Inspiratory muscle warm-up has no impact on performance or locomotor muscle oxygenation during high intensity intermittent sprint cycling exercise. SpringerPlus.

[16] Johnson M.A., Gregson I.R., Mills D.E., Gonzalez J.T., Sharpe G.R. (2014). Inspiratory muscle warm-up does not improve cycling time trial perfomance. Eur. J. Appl. Physiol..

[17] Hawkes E.Z., Nowicky A.V., McConnell A.K. (2007). Diaphragm and intercostal surface EMG and muscle performance after acute inspiratory muscle loading. Respir. Physiol. Neurobiol..

[18] Lomax M., Grant I., Corbett J. (2011). Inspiratory muscle warm-up and inspiratory muscle training: Separate and combined effects on intermittent running to exhaustion. J. Sports Sci..

[19] Stone M.R., Thomas K., Wilkinson M., Gibson A.S.C., Thompson K.G. (2011). Consistency of perceptual and metabolic responses to a laboratory-based simulated 4000-m cycling time trial. Eur. J. Appl. Physiol..

[20] Borg G. (1998). Borg’s Perceived Exertion and Pain Scales.

[21] Wen A., Marlyn M., Woo S., Keens T. (1997). How many maneuvers are required to measure maximal inspiratory pressure accurately?. Chest.

[22] Hopkins W.G., Marshall S.W., Batterham A.M., Hanin J. (2009). Progressive statistics for studies in sports medicine and exercise science. Med. Sci. Sports Exerc..

[23] Faghy M.A., Brown P.I. (2017). Whole body active warm up and inspiratory muscle warm up do not improve running performance when carrying thoracic loads. Appl. Physiol. Nutr. Metab..

[24] Callegaro C.C., Ribeiro J.P., Tan C.O., Taylor J.A. (2011). Attenuated inspiratory muscle metaboreflex in endurance-trained individuals. Respir. Physiol. Neurobiol..

[25] Lomax M., Massey H.C., House J.R. (2017). Inspiratory Muscle Training Effects on Cycling During Acute Hypoxic Exposure. Aerosp. Med. Hum. Perform..

[26] Siebenmann C., Lundby C. (2015). Regulation of cardiac output in hypoxia. Scand. J. Med. Sci. Sports.

[27] Gonçalves T.R., Soares P.P.D.S. (2021). Positive Pressure Ventilation Improves Exercise Performance and Attenuates the Fall of Postexercise Inspiratory Muscular Strength in Rower Athletes. J. Strength Cond. Res..

