# Supplementary material for: The Relationship between Inspiratory Muscle Strength and Cycling Performance: Insights from Hypoxia and Inspiratory Muscle Warm-Up
Source: J Funct Morphol Kinesiol. 2024 May 31;9(2):97. doi: 10.3390/jfmk9020097 (PMC11205061; doi:10.3390/jfmk9020097)
Supplement: Supplementary file 1 [file jfmk-09-00097-s001.zip › jfmk-2994345-supplementary.pdf]

Supplementary Materials

Table S1. Shapiro-Wilk Test p-values results.

Variable	NOR_{PLA}	NOR_{IMW}	HYP_{PLA}	HYP_{IMW}
Elapsed time (s)	0.9858	0.5883	0.4150	0.9773
ΔMIP (%)	0.1532	0.8588	0.2423	0.1993
PO (W)	0.8066	0.1784	0.3784	0.1527
VE (L/min)	0.9455	0.4061	0.9863	0.8385
Borg 6-20	0.5033	0.3994	0.6233	0.0701
Borg CR-10	0.0565	0.6083	0.0033	0.0507
HR (bpm)	0.2880	0.1685	0,2249	0.4361
VS (ml)	0.1599	0.9846	0.6456	0.1129
CO (L/min)	0.2847	0.8200	0.8279	0.2331
SpO_2 (%)	0.1530	0.3561	0.7318	0.3236
MIP (cmH_2O)	0.7398	0.4268	0.3515	0.3223

The results of the normality test. When the '*p*-value' is higher than 0.05, the data is normally distributed. More than 95% of the data was normally distributed.
